# Supplementary material for: Controlling the coherence of a diamond spin qubit through its strain environment
Source: Nat Commun. 2018 May 22;9:2012. doi: 10.1038/s41467-018-04340-3 (PMC5964250; doi:10.1038/s41467-018-04340-3)
Supplement: Supplementary file 1 — Supplementary Information [file 41467_2018_4340_MOESM1_ESM.pdf]

Supplementary material for: Controlling the coherence of a diamond spin qubit through its strain environment

Sohn et al.

1 Supplementary Methods

1.1 Fabrication

An illustration of the oxygen-plasma assisted ion-milling process process for angled etching, and the resulting suspended structure is schematically shown in Supplementary Figure 1(a). The etching occurs over a period of a few hours, during which the stage is rotated constantly while maintaining a fixed angle with the impinging plasma. Further discussion of angled etching techniques can be found in [1, 2]. The focused ion beam (FIB) implantation procedure to generate SiV centers is discussed in detail in [3, 4]. We use a beam energy of 75 keV, predicted to yield an implantation depth of 50 nm±10 nm according to SRIM simulations. The spot size of the ion-beam on the sample is 40 nm, and is expected to determine the lateral precision of the implantation procedure. With regards to conversion efficiency, we implant approximately 50 Si^+ ions per target spot on the sample, and typically generate 1-3 SiVs at each spot after annealing.

After FIB implantation, the sample is then subjected to a tri-acid clean (1:1:1 sulfuric, perchloric, and nitric acids), and a three-step high-temperature high-vacuum annealing procedure [5, 6] in an alumina tube furnace. The annealing sequence followed comprises steps at 400°C (1.5°C per minute ramp, 8 hour dwell time), 800°C (0.5°C per minute ramp, 12 hour dwell time), and 1100°C (0.5°C per minute ramp, 2 hour dwell time). During the entire procedure, the pressure is maintained below 5×10^{-7} torr. Annealing generates a small amount of graphite on the diamond surface, which is subsequently etched away by a tri-acid clean. Following this step, we perform a cleaning in piranha solution to ensure a high level of oxygen-termination at the diamond surface.

Fabrication steps for patterning the metal electrodes are schematically shown in Supplementary Figure 1(b). Here, the triangle represents the cantilever, and the pedestal to the right of the triangle is the location of the bonding pad for electrical contact. The bi-layer PMMA process is repeated twice - first, to define the bonding pad, and second, to define the electrode pattern near the cantilever. This is because, we use a 200 nm thick gold layer for the bonding pad, but only a 10 nm thick tantalum (Ta) layer for the cantilever electrodes. Supplementary Figure 1(c) shows the scheme to connect electrodes on top of the cantilevers to the bonding pad on the diamond pedestal. Electrodes on the substrate below the cantilevers are connected to a second bonding pad (now shown in the figure) that is directly on the surface of the diamond.

We now discuss the choice of 10 nm tantalum film for our cantilever electrodes. For five different metals tested as cantilever electrode material (aluminium, chromium, copper, titanium and tantalum), our device always shows a continuous, non-zero leakage-current upon applying voltage. While the exact reasons for this leakage-current on our sample, in particular, are unknown, there have been numerous studies on the surface conductivity of diamond under various conditions [7]. For aluminium, chromium, copper and titanium, this leakage current destroys the electrode when a high voltage (in the few hundred volts range) is applied. The destruction of electrodes appeared to be the result of melting or bursting of

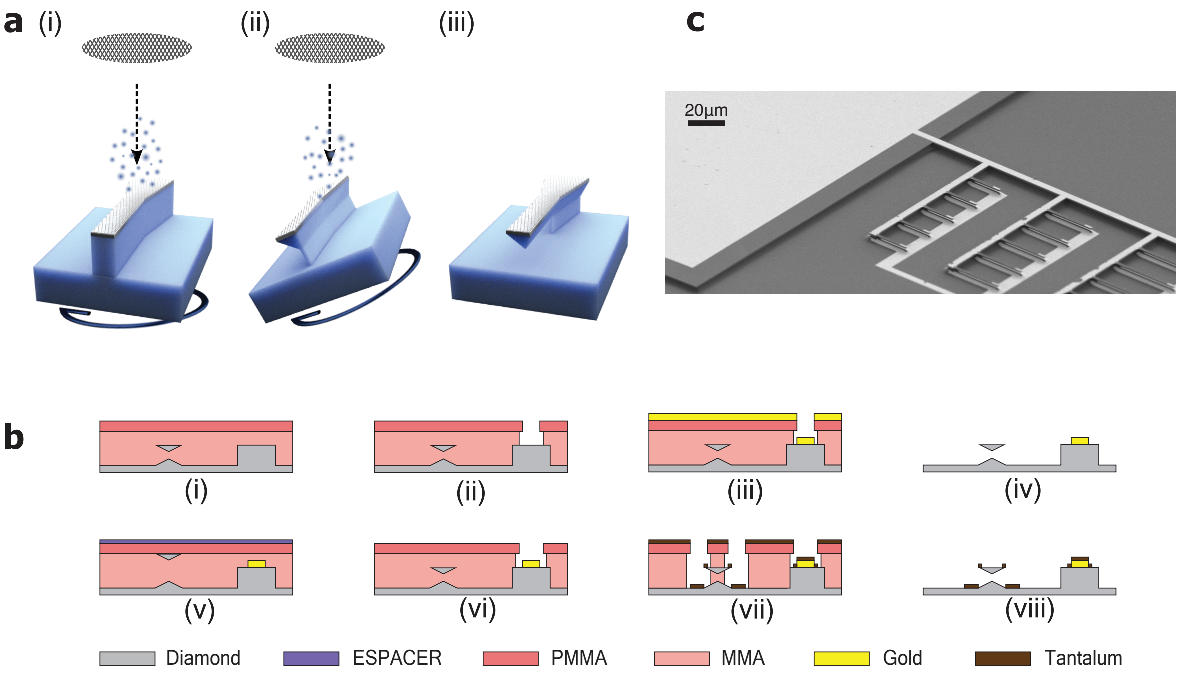

Supplementary Figure 1: (a) Schematic of oxygen-plasma assisted ion-milling process for angled-etching of diamond cantilevers. The ion beam is directed at the diamond sample, with a vertically-etched device pattern. The tilted stage is continuously rotated during the etching process. After the cantilevers are freely standing, the etch-mask is stripped. (b) Fabrication process for the placement of electrodes. First, the coarsely aligned bonding pad is defined with a bi-layer PMMA process followed by gold evaporation. Then the same process is repeated to define tantalum electrodes near cantilevers, but with better alignment precision. Conductive layer (ESPACER 300Z) on top of the cantilever is helpful for precise alignment. (c) SEM image of the complete chip showing connection between the bonding pad and electrodes on top of the cantilevers.

the thin metal film, likely caused by Joule heating [8]. Tantalum is one of the metals with the highest melting and evaporation points among those available for e-beam evaporation. We find that devices with tantalum electrodes are robust enough to operate at very high applied voltage ($\sim$600 V across an electrode gap of approximately 4 μm, which corresponds to an electric-field of 1.5 MV/cm). The thickness of tantalum is kept below 10 nm in order to avoid thin film-induced stress in the cantilever, which leads to pre-strained SiV centers.

1.2 Device design

An important figure of merit for our NEMS device is the maximum achievable strain at the location of SiV. In this section, we discuss two key design aspects that need to be considered towards this goal: (i) ‘pull-in instability’, and (ii) practical limits for high voltage operation.

Pull-in instability is a well-known phenomenon for an electrostatic actuator made with a

parallel plate capacitor as shown in Supplementary Figure 2(a). In these devices, voltage is applied to induce an electrostatic force between two plates, where either one or both of them are free to move. Upon applying a voltage, the capacitor deforms until it reaches equilibrium, when there is a balance between the electrostatic force, and the restoring force exerted by the elasticity of the material. The net force acting on the free top plate in Supplementary Figure 2(a) can be modeled as

$$F(x,V) = -\frac{\partial U(x,V)}{\partial x} = \frac{1}{2}\varepsilon A \frac{V^2}{(d-x)^2} - kx \tag{1}$$

where x is the displacement of the plate, $U(x,V)$ is the potential energy and ε is the permittivity of the material between the two plates. A is the area of the capacitor, V is the voltage applied, d is the distance between the two plates at 0 V, and k is the spring constant, respectively. By integrating Supplementary Equation 1, we can calculate $U(x,V)$ at various voltages as shown in Supplementary Figure 2(b). The local minimum in the potential represents a condition of stable equilibrium. As the voltage is increased, the local minimum shifts towards the bottom plate, indicating that the top plate gets displaced downwards, thereby reducing the capacitor gap. When the voltage changes from $3V_0$ to $4V_0$ in the Supplementary Figure 2(b), the stable local minimum disappears. This occurs, when the top plate is displaced by about one-third of the initial gap between the two plates, i.e. when $x = d/3$. At this point, the system reaches a condition in which the top plate snaps down to the bottom plate. Our device is a slight variation of this conceptual model, and hence, the maximum deflection of our cantilever will be limited by pull-in instability (but not at exactly $x = d/3$).

A 3D finite element method (FEM) calculation can be used to simulate pull-in instability accurately for complex structures [9]. Typically, a simulation can be run by setting a particular voltage on the electrode, and solving for the resultant deformation of the structure, thereby arriving at the strain profile inside the cantilever. One can also run the inverse of this procedure. By setting a target displacement of the cantilever tip, the voltage required to achieve this displacement can be calculated. Such an inverse calculation can help arrive at the condition for pull-in instability. Supplementary Figure 2(c) shows the results of a simulation run so as to solve this inverse problem. Strain at the SiV location is plotted as a function of the voltage for different cantilever lengths. Turnaround points represent the pull-in instability condition at which both the displacement of the cantilever-tip and the applied voltage reach the maximum value possible before the cantilever snaps down. Supplementary Figure 2(c) provides two important conclusions: First, for a given voltage, longer cantilevers provide larger strain for a given voltage, because they have a smaller spring constant. Second, the maximum attainable strain is higher for shorter cantilevers, because they reach the pull-in instability condition at a higher voltage. Therefore, shorter devices are preferred to generate high strain, when arbitrarily high voltage can be applied.

In practice, however, there are mechanisms that limit the maximum possible voltage e.g. Townsend breakdown, field emission and surface current [10, 11, 12], all of which can be significant depending on experimental conditions. With the fabrication method described in Section 1.1, our devices could be operated safely up to voltage as high as 600 V under high vacuum ($\sim 10^{-7}$ torr) at cryogenic temperature (4 K). Given that the minimum electrode

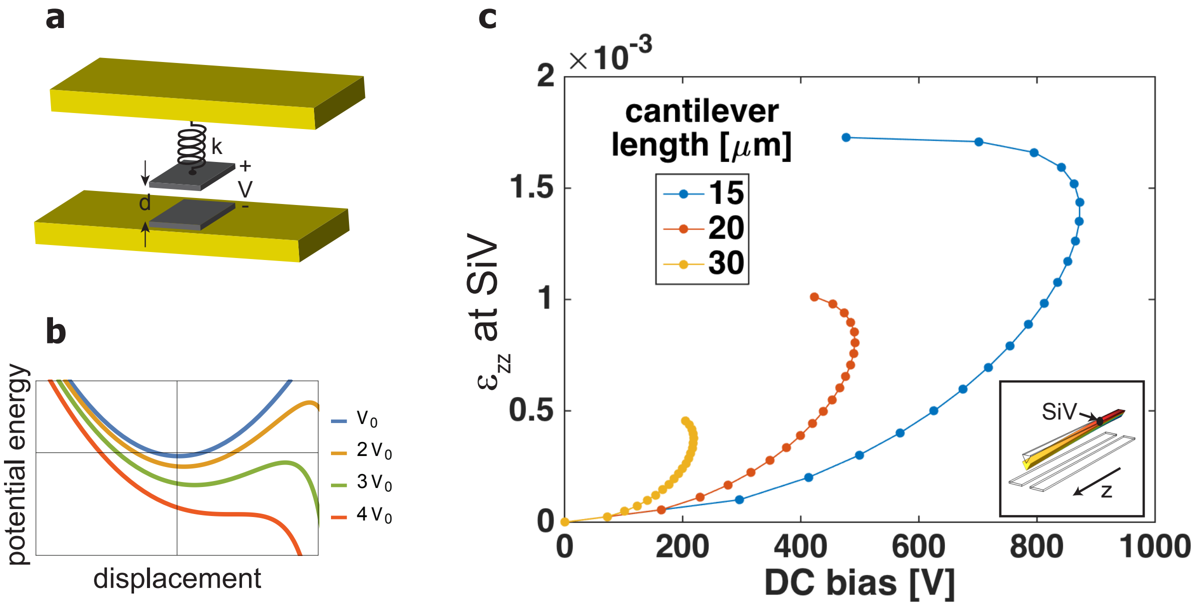

Supplementary Figure 2: (a) Illustration of a parallel plate capacitor with one freely movable plate (top), and one fixed plate (bottom). The top electrode can be actuated by applying a voltage. (b) Potential energy of the system in (a) with the different voltages. The stable minimum in the potential disappears, when the system reaches the condition of pull-in instability at a voltage of $4V_o$. (c) FEM simulation of the strain-component along the long-axis of the cantilever (most dominant strain-tensor component) near the clamp of the cantilever (inset). Turnaround points in the graph represent pull-in instabilities.

gap is $4\,\mu$m, this condition corresponds to an electric field of approximately 1.5 MV/cm. The coherent population trapping (CPT) experiments described in the main text are carried out in a helium closed-cycle cryostat with the sample surrounded by helium exchange gas at a pressure of 1 mbar. Under these conditions, we observed safe operation up to 500 V. The maximum voltage in this setup is thought to be limited by dielectric breakdown of helium gas.

Considering all the design limitations discussed above, we chose cantilevers of width 1.2-1.3 μm and length 25-30 μm for the experiments in this work.

1.3 Strain-dependent photoluminescence measurements

Strain-response measurements involve taking resonant excitation spectra as the voltage applied to the device is steadily increased. Such a sequence of spectra is shown in Supplementary Figure 7, and is used to derive Fig. 2a in the main text. Strain corresponding to the applied voltage is estimated through finite element method (FEM) simulations [13].

1.4 Orbital thermalization measurements

We use time resolved pump-probe fluorescence to characterize the phonon processes in the GS. In this method, two consecutive laser pulses resonant with the D transition are used to, first initialise GS orbital population in the lower branch $|1\rangle$, and after a set delay τ, read-out population in the upper branch $|2\rangle$. A schematic of the pulse sequence, and an example of a resulting fluorescence time-trace are shown in Supplementary Figure 3. By repeating this sequence for steadily increasing pump-probe delay τ, we measure the rate at which the GS population relaxes towards thermal equilibrium due to resonant phonons.

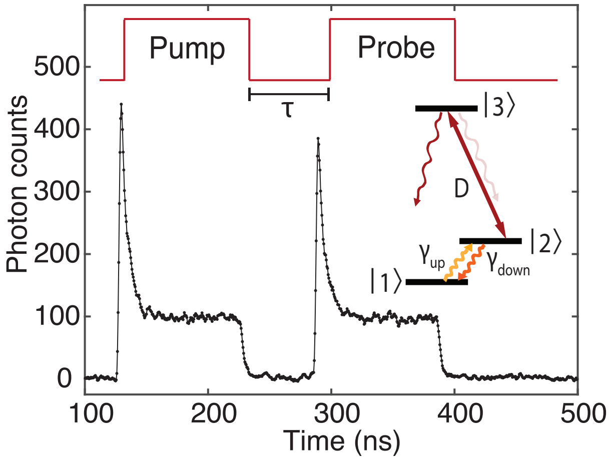

Supplementary Figure 3: Time-resolved fluorescence signal in pump-probe measurement for a delay $\tau = 50$ ns between the two laser pulses. The laser is resonant with the D transition, and optically pumps the GS population into the lower orbital branch $|1\rangle$ over a timescale of few ns. After time τ, the fluorescence signal from the probe pulse has a leading edge determined by the population in the upper orbital branch $|2\rangle$. The decay rates between levels $|1\rangle$ and $|2\rangle$ - γ_{up} due to phonon-absorption, and γ_{down} due to phonon-emission - are also shown.

Extraction of thermalization rate

Example data from implementing the pulse sequence in Supplementary Figure 3 for various pump-probe delays is shown in Supplementary Figure 4. This data can be interpreted and processed to yield a GS-population thermalization curve as follows. As shown in Supplementary Figure 5a, the leading edge of the first fluorescence signal corresponds to thermal population $p_{2,\mathrm{th}}$ in the GS level $|2\rangle$. Upon switching on the pump pulse, this decays to a residual value $p_{2,\mathrm{opt}}$ determined by the competition between the optical pumping rate (above saturation, this is simply the decay rate γ_e from the excited state $|3\rangle$) and the rates $\gamma_{\mathrm{up}}, \gamma_{\mathrm{down}}$. After time delay τ, the leading edge of the probe fluorescence signal corresponds to partially recovered population $p_2(\tau)$ due to thermalization. We can describe the population recovery in level $|2\rangle$ as

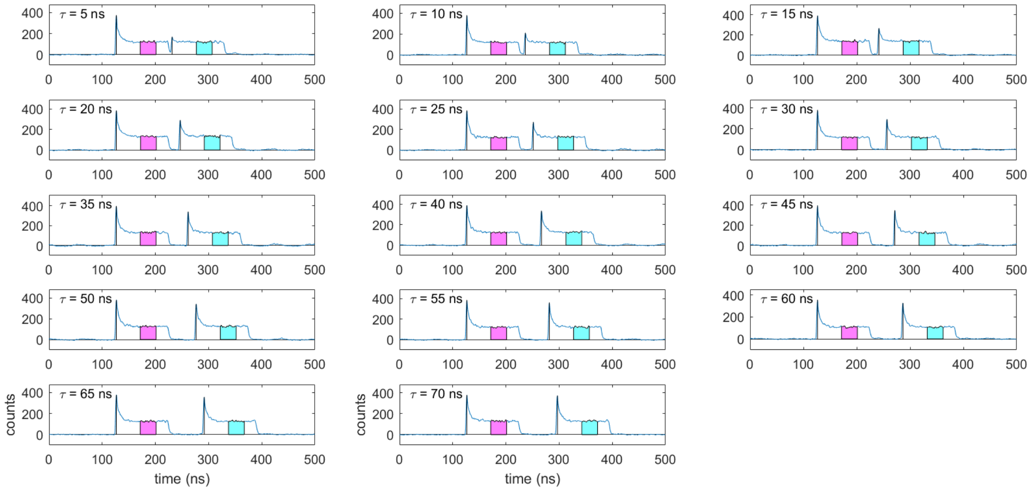

Supplementary Figure 4: Fluorescence time-traces for various pump-probe delays between τ=5 ns to 70 ns taken at GS-splitting $\mathit{\Delta}_{\mathrm{gs}}$=46 GHz. x-axis is time in ns, and y-axis is photon counts integrated over multiple iterations of the pulse sequence.

$$(p_2(\tau) - p_{2,\mathrm{th}}) = (p_2(\tau) - p_{2,\mathrm{opt}}) \exp\left[-\left(\gamma_{\mathrm{up}} + \gamma_{\mathrm{down}}\right) t\right] \tag{2}$$

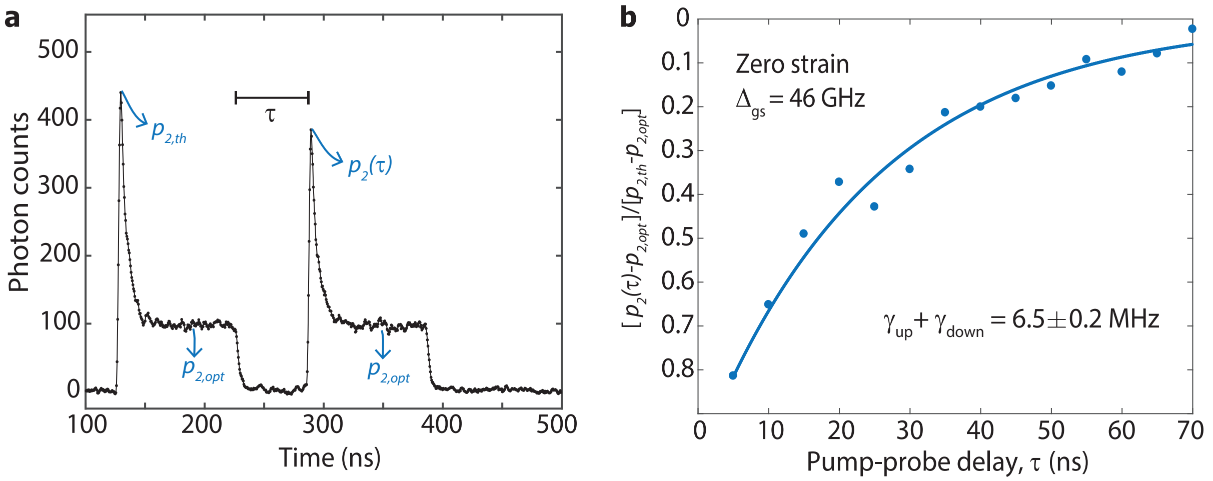

Supplementary Figure 5: (a) Fluorescence time-trace for τ=50 ns from Supplementary Figure 4 showing relevant quantities related to the population in level $|2\rangle$. (b) Thermalization curve constructed by extracting the normalized change in photon-counts for various pump-probe delays τ. Solid line is an exponential fit.

In particular, we calculate the normalized change in photon-counts, $(p_2(\tau) - p_{2,\mathrm{th}}) \, / \, (p_2(\tau) - p_{2,\mathrm{opt}})$ from each measurement in Supplementary Figure 4, and carry out an exponential fit in Supplementary Figure 5b to extract $(\gamma_{\mathrm{up}} + \gamma_{\mathrm{down}})$. Repeating this experiment for various values

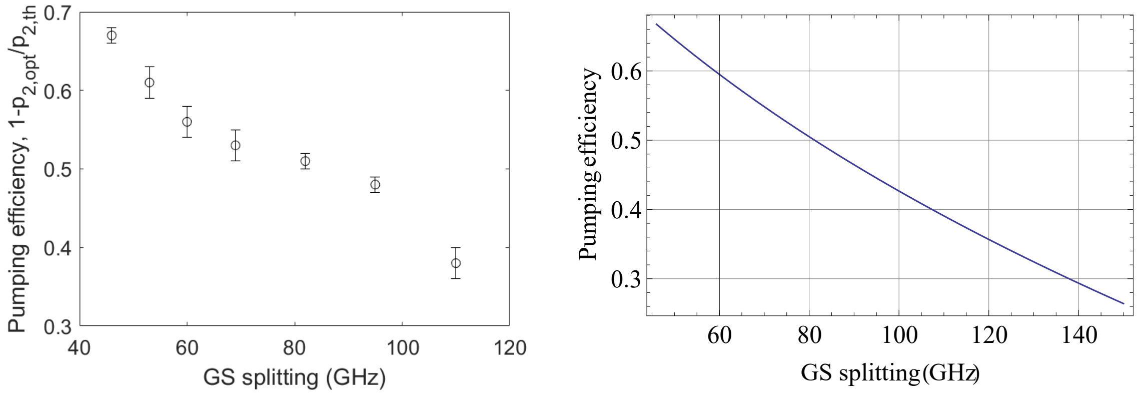

Supplementary Figure 6: (a) Experimentally measured optical-pumping efficiency (see text for definition) versus GS-splitting $\Delta_{\rm gs}$. (b) Theoretically estimated optical-pumping efficiency using a three-level rate equations model. We assume optical pumping above saturation, GS thermalization rates, $\gamma_{\rm up}$ and $\gamma_{\rm down}$ to follow the model in Section 2.2, excited state decay rate $\gamma_e = 90$ MHz, branching ratio into $|1\rangle = 0.5$.

of GS-splitting $\Delta_{\rm gs}$, we arrive at Fig. 2c in the main text.

From the optical-pumping fluorescence signal, we can define a pumping efficiency $\left(1 - \frac{p_{2,\rm opt}}{p_{2,\rm th}}\right)$. This describes the ability of the pump pulse to initialize GS population in level $|1\rangle$ by acting against the thermalization process. As the GS-splitting $\Delta_{\rm gs}$ is increased, we find that the pumping-efficiency decreases in Supplementary Figure 6. This can be attributed to the downward thermalization rate $\gamma_{\rm down}$ rapidly increasing with $\Delta_{\rm gs}$ as will be discussed in Section 2.2. At large $\Delta_{\rm gs}$, the optical pumping rate, $R_{\rm opt}$, which is $\sim \gamma_{\rm e}$, the excited state decay rate cannot substantially outweigh $\gamma_{\rm down}$. As a result, the GS population cannot be optically pumped into level $|1\rangle$ better than is dictated by thermal equilibrium. This makes it impractical to measure the thermalization rate beyond a certain $\Delta_{\rm gs}$ using the pump-probe technique. In our experiments, we measure up to $\Delta_{\rm gs} = 110$ GHz.

2 Supplementary Discussion

2.1 High-strain PLE spectra

In our devices, we can generate uniaxial strain as high as $\sim 10^{-3}$ at a voltage of 600 V. Example spectra showing strain-tuning into this regime are shown in Supplementary Figure 8. At high strain, as $\Delta_{\rm gs} \gg k_B T \sim$80 GHz at T=4 K, population in the upper branch $|2\rangle$ of the ground-state manifold decreases exponentially, while the population in the lower branch $|1\rangle$ correspondingly increases to near-unity. As a result, with increasing strain, the B and D transitions become weaker in intensity, and eventually vanish. Simultaneously, the linewidth of the A transition increases owing to increasingly rapid phonon-mediated relaxation in the excited state from $|4\rangle$ to $|3\rangle$. This is captured by the model for single-phonon emission and absorption discussed in Section 2.2. On the other hand, as the C transition connects the

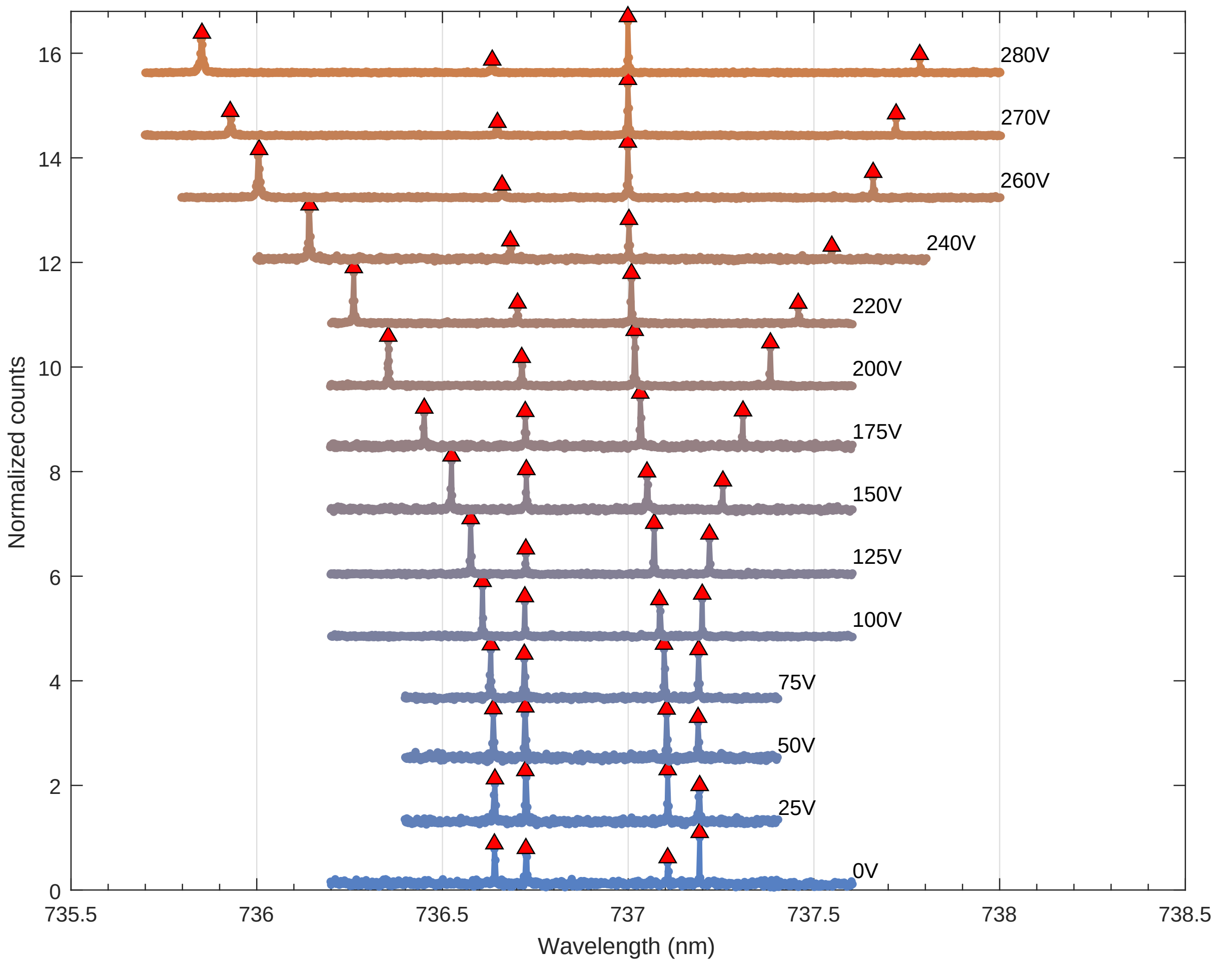

Supplementary Figure 7: PLE spectra of an SiV center with increasing voltage applied to the device

lower orbitals in ground and excited states ($|1\rangle$ and $|3\rangle$), its linewidth is not affected as strongly by phonon-mediated processes, and is measured to be relatively unchanged with strain (See Section 2.3). The C transition also becomes brighter at high-strain due to near-unity population in the lower ground state branch $|1\rangle$.

While the high-strain spectra only reveal A and C transitions, the difference in their frequencies gives us the excited-state splitting Δ_{es} exactly. Since the same strain-components are responsible for increasing Δ_{gs} and Δ_{es}, we can use the strain-susceptibilities in [14, 13] to estimate Δ_{gs}. Using this procedure, for the highest strain condition in Supplementary Figure 8, we infer $\Delta_{\mathrm{gs}} = 1.2\,\mathrm{THz}$. A more precise experimental technique to measure the GS- and ES-splittings at high strain is described in Section 2.2.

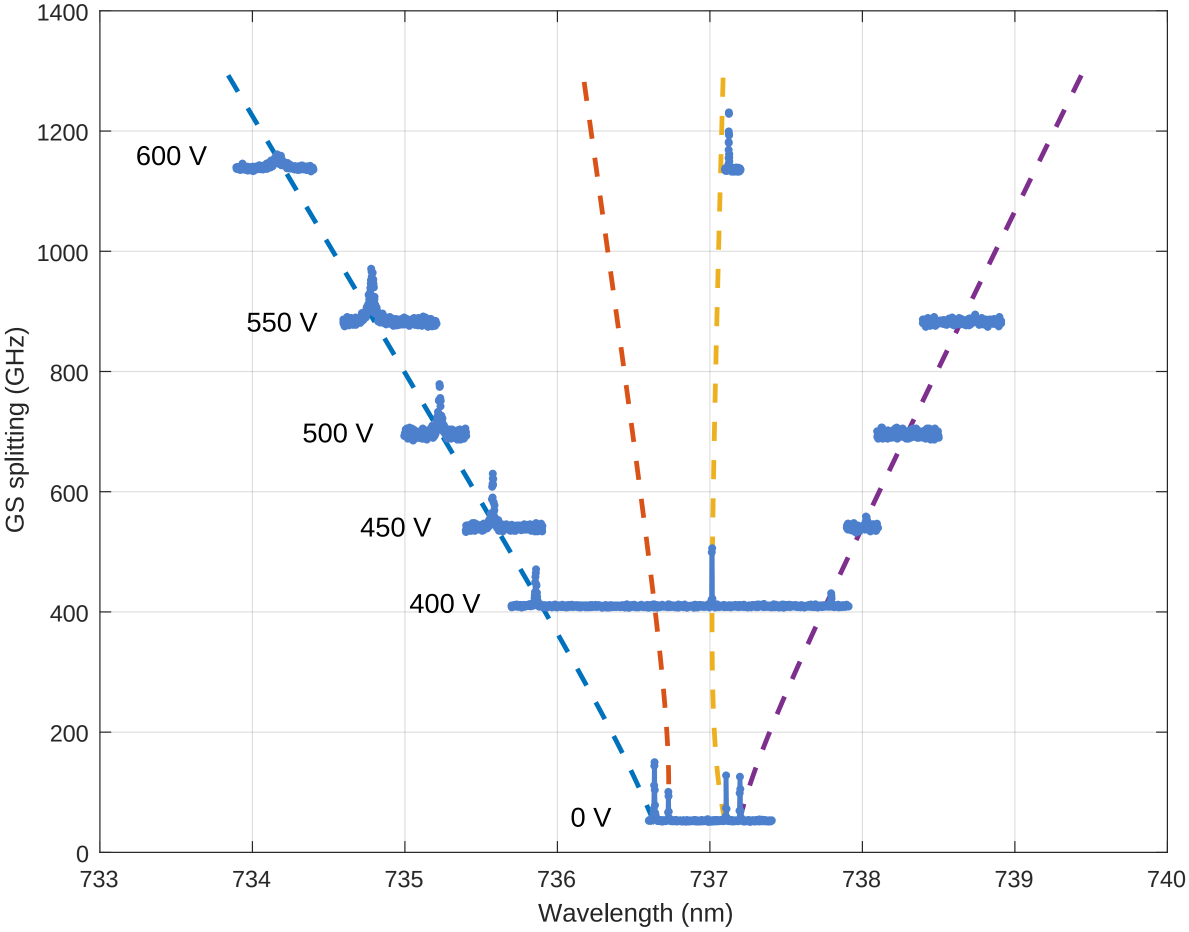

Supplementary Figure 8: Tuning of PLE spectrum of a transverse SiV subject to high strain. Voltage applied to the device is indicated next to each spectrum. y-axis shows ground-state splitting Δ_{gs} corresponding to the spectrum estimated using the procedure detailed in the text. Dashed lines correspond to modeled strain-response of the four optical transitions.

2.2 Orbital thermalization model

Theory

Due to the nature of the strain interaction, phonons that are resonant with the ground state splitting Δ_{gs} can directly drive the orbital transition [15]. The transition rates driven by the continuum of lattice phonon modes can be calculated using Fermi's golden rule [16] to get:

$$\gamma_{\text{up}}(\Delta_{\text{gs}}) = 2\pi\chi\rho\Delta_{\text{gs}}^3 n_{\text{th}}(\Delta_{\text{gs}}) \tag{3}$$

$$\gamma_{\text{down}}(\Delta_{\text{gs}}) = 2\pi\chi\rho\Delta_{\text{gs}}^3 (n_{\text{th}}(\Delta_{\text{gs}}) + 1) \tag{4}$$

where ρ is a constant proportional to average speed of sound in bulk, n_{th} is the number of thermal phonons per mode and χ is the interaction frequency for a single phonon. In these

expressions, the first term in the product, $2\pi\chi\rho\Delta_{\text{gs}}^3$ corresponds to the mean-squared single-phonon coupling rate multiplied with the DOS at the GS splitting Δ_{gs}, while the second term corresponds to the thermal occupation of each mode. Supplementary Figure 9 shows plots of the calculated dependence of upward and downward rates on Δ_{gs} at temperature $T = 4\,\text{K}$. We observe that the upward rate shows a non-monotonic behavior, approaching its maximum value around $h\Delta_{\text{gs}} \sim k_B T$. The increasing DOS term dominates in the regime $h\Delta_{\text{gs}} < k_B T$, and causes γ_{up} to increase. However, when $h\Delta_{\text{gs}} \gg k_B T$, the thermal occupation of the modes behaves as $n_{\text{th}}(\Delta_{\text{gs}}) = \exp\left(-\frac{h\Delta_{\text{gs}}}{k_B T}\right)$. This exponential roll-off dominates the polynomially increasing DOS, and causes γ_{up} to decrease at higher strain. In contrast, the downward rate monotonically increases with the GS-splitting, because it is dominated by the spontaneous emission rate, which simply scales as the DOS.

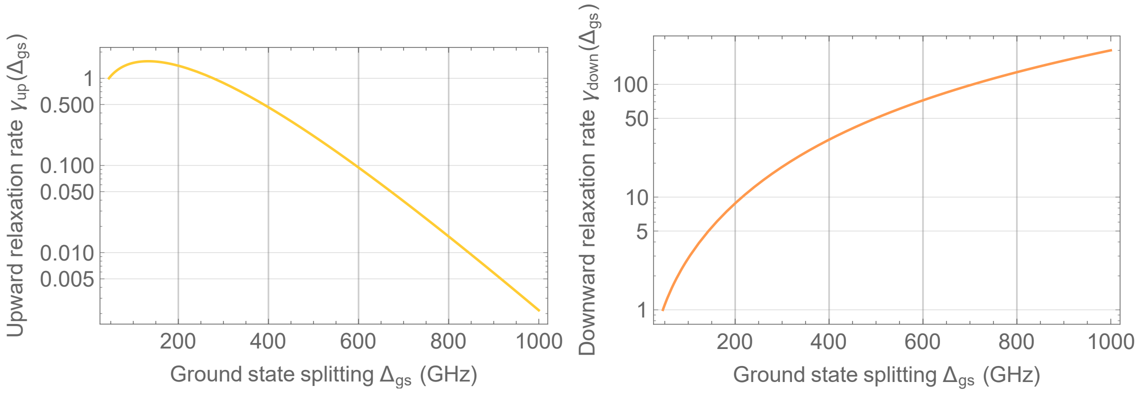

Supplementary Figure 9: Variation of the upward (phonon-absorption) and downward (phonon-emission) relaxation rates with GS-splitting Δ_{gs} at temperature T=4 K. The y-axis is normalized to the zero-strain rates $\gamma_{\text{up}}(46\text{GHz})$ and $\gamma_{\text{down}}(46\text{GHz})$ in each plot respectively.

Fitting

With the pump-probe technique used in our experiments, we measure the sum of the upward and downward rates, which should have the dependence

$$\gamma_{\text{up}}(\Delta_{\text{gs}}) + \gamma_{\text{down}}(\Delta_{\text{gs}}) = A\Delta_{\text{gs}}^3(2n_{\text{th}}(\Delta_{\text{gs}}) + 1) = A\Delta_{\text{gs}}^3 \coth\left(\frac{h\Delta_{\text{gs}}}{k_B T}\right) \tag{5}$$

where A is a constant. Our cantilever has lateral dimensions of order $1\,\mu$m. This is about an order of magnitude larger than half the acoustic wavelength for $\Delta_{\text{gs}} = 50\,\text{GHz}$ in diamond, which is $\sim$120 nm. Thus, the cantilever itself is a bulk-like structure for the SiV. However, as can be observed in the left panel of Supplementary Figure 10 our experimental data shows very poor agreement with the theoretically predicted behavior in Supplementary Equation 5.

Instead, when we allow the exponent of the DOS term to be a variable n, we obtain a better fit yielding $n = 1.9 \pm 0.3$. We can arrive at a possible explanation for this discrepancy, if we note that the SiV is situated at a nominal depth of 50 nm, which is sub-wavelength for

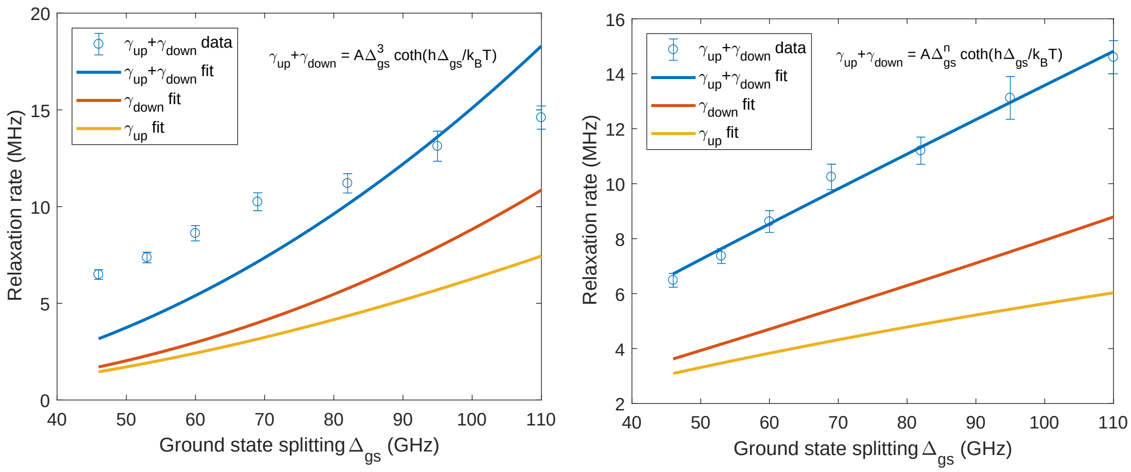

Supplementary Figure 10: Fits to experimental measurements of net orbital thermalization rate $\gamma_{\mathrm{up}} + \gamma_{\mathrm{down}}$ (solid blue line) assuming coupling to only bulk acoustic modes (left plot), and unknown DOS behavior (right plot) with varying n. Fit expressions are indicated in top left corner of each plot. Corresponding individual rates γ_{up} and γ_{down} are extracted and plotted after fitting in each case (solid yellow and orange lines). The fit to variable DOS (right plot) yields $n = 1.9 \pm 0.3$.

phonons in the frequency range of Δ_{gs} probed in our experiment. As a result, we can expect appreciable coupling to surface acoustic modes, which are not accounted for in the above derivation. Since the DOS of surface modes scales as Δ_{gs}^2, it appears that the thermalization rate in our experiment is almost entirely determined by surface modes.

Calibration of ground and excited state splittings with applied voltage at high strain

The ground state splitting can be measured directly by considering the energy difference between the transitions labelled C and D in Fig. 2(a) of the main text. Due to the presence of other SiV^- centres generated at the same spot due to the FIB implantation procedure, measuring this energy difference between C and D transitions through non-resonant PL spectra is impractical. At the same time, simultaneous detection of C and D transitions through resonant excitation is not possible at high strain, since the Boltzmann population in level $|2\rangle$ becomes negligible as discussed in Section 2.1. To overcome this limitation, we resonantly excite transition A of the SiV center being studied, and record the spectrum of transitions C and D on a spectrometer after having filtered out the resonant laser with a monochromator. The spectra are then fitted with two Lorentzian functions to extract the value of the ground state splitting, as shown in Supplementary Figure 11. Likewise, the excited state splitting (also shown in the figure) can be derived as the difference between the frequencies of transitions A and C in this measurement.

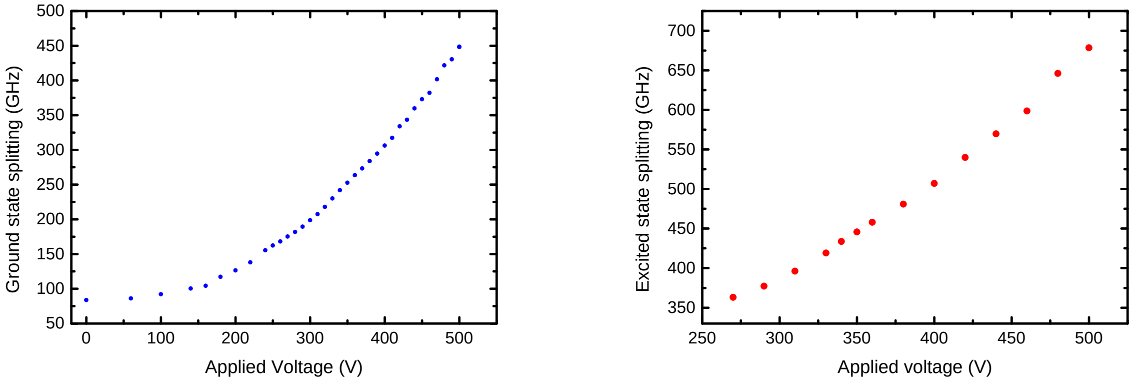

Supplementary Figure 11: SiV ground state splitting (left), and excited state splitting (right) measured as a function of voltage applied to the cantilever. Error bars are smaller than the dots and correspond to the standard deviation on the frequency difference between C and D transitions estimated from Lorentzian fits.

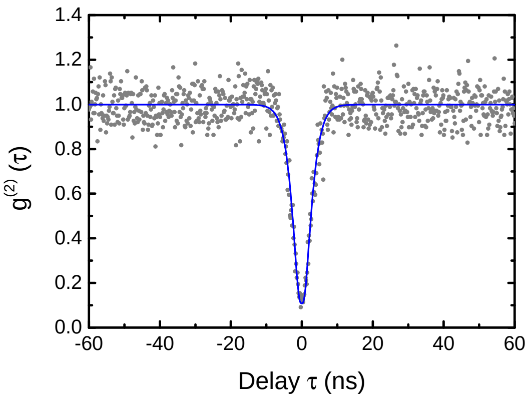

Supplementary Figure 12: Second-order correlation measurement of the SiV^- centre investigated. The measured data points are plotted as grey dots. A fit based on a three-level model and accounting for timing jitter is plotted as a blue curve.

2.3 Investigation of double-dip CPT signal

We first rule out the hypothesis that the two dips in our CPT measurements originate from two different SiV centres. The dips are very similar in width and depth, and their frequency-separation remains constant (4.0 ± 0.1 MHz) over a wide range of applied strain (see Fig. 4b of the main text). In the event that this is caused by two SiV centers, they are required to have the same fluorescence intensity, and experience exactly the same strain conditions at any applied voltage, which is very unlikely. In order to categorically establish that we are investigating a single SiV centre, we perform a Hanbury-Brown-Twiss (HBT) experiment on the phonon-sideband fluorescence upon resonant excitation of transition C, and measure the second order correlation function $g^{(2)}$ as shown in Supplementary Figure 12. The $g^{(2)}$ function is fitted using a three-level model described in [17] convolved with the Gaussian

response of the avalanche photodiodes used, which have a timing jitter of 350 ps. At zero time delay, a clear anti-bunching reaching $g^{(2)}(\tau = 0) = 0.12$ indicates that the measured photons originate from a single emitter.

To gain more insight into the origin of the two CPT dips, we perform CPT at varying orientation of the applied magnetic field, while keeping its magnitude (0.2 T) constant. We work in the high-strain regime at a ground state splitting of 467 GHz. In our results, shown in Supplementary Figure 13, we observe that the separation between the two CPT dips displays a periodic variation as the magnetic field is rotated.

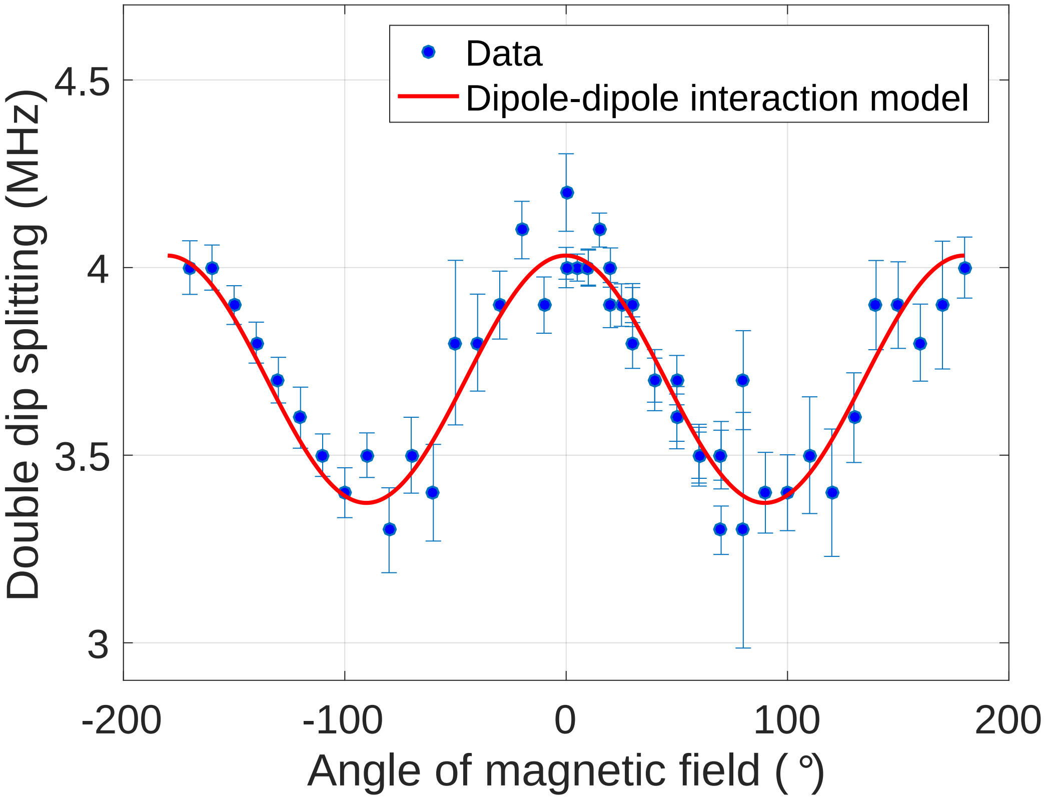

Supplementary Figure 13: Dependence of CPT dip separation on magnetic field orientation. The angle plotted on the x-axis is measured with respect to the vertical direction on the sample. $0°$ corresponds to the [001] axis of diamond, while $90°$ corresponds to the [110] axis of diamond, along which the cantilever long-axis is aligned. The SiV investigated is a transverse SiV, so its internal Z-axis is either $[1\bar{1}1]$ or $[\bar{1}11]$. Error bars correspond to the standard deviation on the CPT dip frequencies estimated from Lorentzian fits.

Given the similarity of the two dips, a very plausible explanation for the double-dip structure is the presence of a proximal spin in the environment of the SiV centre being studied. Phys-

ically, varying the direction of the applied B-field leads to a variation in the quantization axis of the SiV electron-spin (or semi-classically, the orientation of the electron magnetic moment). Likewise, the quantization axis of the proximal spin has its own variation with the B-field orientation. For instance, if this proximal spin is a nuclear spin, to first order, its orientation simply follows that of the applied B-field. As a result, the dipole-dipole interaction energy of the SiV electron-spin with the neighboring spin varies with B-field orientation, leading to the periodic behavior observed experimentally in Supplementary Figure 13. Below, we describe a semi-classical approach to model the CPT dip separation as a dipole-dipole interaction.

The Hamiltonian for two dipoles with magnetic moments $\boldsymbol{\mu_1}$ and $\boldsymbol{\mu_2}$ is given by

$$\mathbb{H}^{\text{d-d}} = -\frac{\mu_0}{4\pi\left|\mathbf{r}\right|^3}\left(3\left(\boldsymbol{\mu_1}\cdot\hat{\mathbf{r}}\right)\left(\boldsymbol{\mu_2}\cdot\hat{\mathbf{r}}\right) - \boldsymbol{\mu_1}\cdot\boldsymbol{\mu_2}\right) \tag{6}$$

where μ_0 is the vacuum-permeability, $\mathbf{r}$ is the vector from one dipole to the other, and $\hat{\mathbf{r}}$ is given by $\mathbf{r}/\left|\mathbf{r}\right|$. If the two dipoles are spins described by spin angular momentum $\mathbf{S}_1$ and $\mathbf{S}_2$, we can write the interaction Hamiltonian in terms of the spin-operators.

$$\mathbb{H}^{\text{d-d}} = -\frac{\mu_0\gamma_1\gamma_2}{4\pi\left|\mathbf{r}\right|^3}\left(3\left(\mathbf{S}_1\cdot\hat{\mathbf{r}}\right)\left(\mathbf{S}_2\cdot\hat{\mathbf{r}}\right) - \mathbf{S_1}\cdot\mathbf{S_2}\right) \tag{7}$$

where γ_i is the gyromagnetic ratio of spin i. Semi-classically, we can treat spin angular momentum as a vector quantity $\mathbf{S} = \frac{\hbar}{2}\left(\langle\sigma_x\rangle, \langle\sigma_y\rangle, \langle\sigma_z\rangle\right)$, where $\sigma_x, \sigma_y, \sigma_z$ are Pauli spin-matrices. This vector describes the mean-orientation of the electron magnetic moment. To calculate the SiV electron-spin orientation under our experimental conditions, the full ground-state Hamiltonian including spin-orbit coupling, external strain, and magnetic field must be diagonalized. The effect of the external magnetic field is described by the Zeeman Hamiltonian below written in the basis $\{|e_x,\uparrow\rangle, |e_x,\downarrow\rangle, |e_y,\uparrow\rangle, |e_y,\downarrow\rangle\}$ [18],

$$\mathbb{H}^{\text{Zeeman}} = q\gamma_{\text{L}}\hat{L}_z B_z + \gamma_{\text{S}}\hat{\mathbf{S}}\cdot\mathbf{B} = q\gamma_L\begin{bmatrix} 0 & 0 & iB_z & 0 \\ 0 & 0 & 0 & iB_z \\ -iB_z & 0 & 0 & 0 \\ 0 & -iB_z & 0 & 0 \end{bmatrix} + \gamma_S\begin{bmatrix} B_z & B_x - iB_y & 0 & 0 \\ B_x + iB_y & -B_z & 0 & 0 \\ 0 & 0 & B_z & B_x - iB_y \\ 0 & 0 & B_x + iB_y & -B_z \end{bmatrix} \tag{8}$$

where the first and second terms are from the orbital angular momentum, and the spin angular momentum, respectively. $\hat{L}_z$ and $\hat{S}$ are L_z and $\mathbf{S}$ normalized by $\hbar$, respectively. The gyromagnetic ratios for each term are given by $\gamma_{\text{L}} = \mu_{\text{B}}/\hbar$, $\gamma_{\text{S}} = 2\mu_{\text{B}}/\hbar$, where μ_{B} is the Bohr magneton. q is a quenching factor that is commonly observed in solid-state emitters [15].

The total Hamiltonian $\mathbb{H}^{\text{total}}$ is obtained by adding the spin-orbit Hamiltonian $\mathbb{H}^{\text{SO}}$ and strain Hamiltonian $\mathbb{H}^{\text{strain}}$.

$$\mathbb{H}^{\text{total}} = \mathbb{H}^{\text{SO}} + \mathbb{H}^{\text{strain}} + \mathbb{H}^{\text{Zeeman}} \tag{9}$$

$$\mathbb{H}^{\text{SO}} + \mathbb{H}^{\text{strain}} = \begin{bmatrix} \alpha-\beta & 0 & \gamma - i\lambda_{\text{SO}}/2 & 0 \\ 0 & \alpha-\beta & 0 & \gamma + i\lambda_{\text{SO}}/2 \\ \gamma + i\lambda_{\text{SO}}/2 & 0 & \alpha+\beta & 0 \\ 0 & \gamma - i\lambda_{\text{SO}}/2 & 0 & \alpha+\beta \end{bmatrix} \tag{10}$$

λ_{SO}, the spin-orbit coupling is 46 GHz for the ground state of the SiV. α, β, γ are strain tensor components obeying the symmetries of the SiV center as defined in [13, 18]. We diagonalize the total Hamiltonian, $\mathbb{H}^{\text{total}}$, and calculate expectation values of the Pauli matrices for the lowest two eigenstates, which comprise the SiV spin-qubit under investigation. This gives us the mean orientation of the SiV electron-spin, say $\mathbf{S}_1$ for given experimental conditions. To calculate the mean orientation of the proximal spin $\mathbf{S}_2$, we assume it to be either a nuclear spin such as ^{13}C, or an electron spin such as another SiV-center. In the case of a nuclear spin, $\mathbf{S}_2$ is simply given by the direction of the external magnetic field. Once the quantization axes of the two spins are known, we can fit our data to the calculated value of $\mathbb{H}^{\text{d-d}}$ from Eq. 7 by using the distance between the spins $\mathbf{r}$ as a fit parameter. The result of such a fitting procedure is shown in Supplementary Figure 13. In the case of a nuclear spin, the distance between the two spins $|\mathbf{r}|$ is estimated to be on the order of 1 Å. If the other spin is an electron spin from another SiV centre, it is possible to obtain similar results as in Supplementary Figure 13. However, in this case, the distance between the spins is on the order of tens of nanometres.

Supplementary Note 1: Spectral diffusion of C transition

The C transition of the SiV centre used in our CPT measurements does not experience extra spectral diffusion at high strain (high voltage). In theory, there are two potential causes of spectral diffusion that might appear at high strain. (i) A gradient of strain could perturb the inversion symmetry of the SiV-center, and induce a nonzero permanent dipole moment for the electronic levels. (ii) A large DC-electric field at the SiV location arising from the applied voltage could induce a linear Stark shift response to fluctuating electric-fields in the environment of the SiV, thereby increasing spectral diffusion. In our experiments, the amount of spectral diffusion on the timescale of minutes (roughly ±20 MHz) does not appear to increase from the zero-voltage to the high-voltage condition (Supplementary Figure 14). This is further confirmed by our measurements of the C transition linewidth at various high strain conditions. (Supplementary Figure 15). We do observe a systematic drift in the transition frequency on the timescale of hours, but this slow drift can be easily corrected for by adjusting the resonant laser excitation frequency (or alternatively by performing feedback on the DC voltage applied to our device).

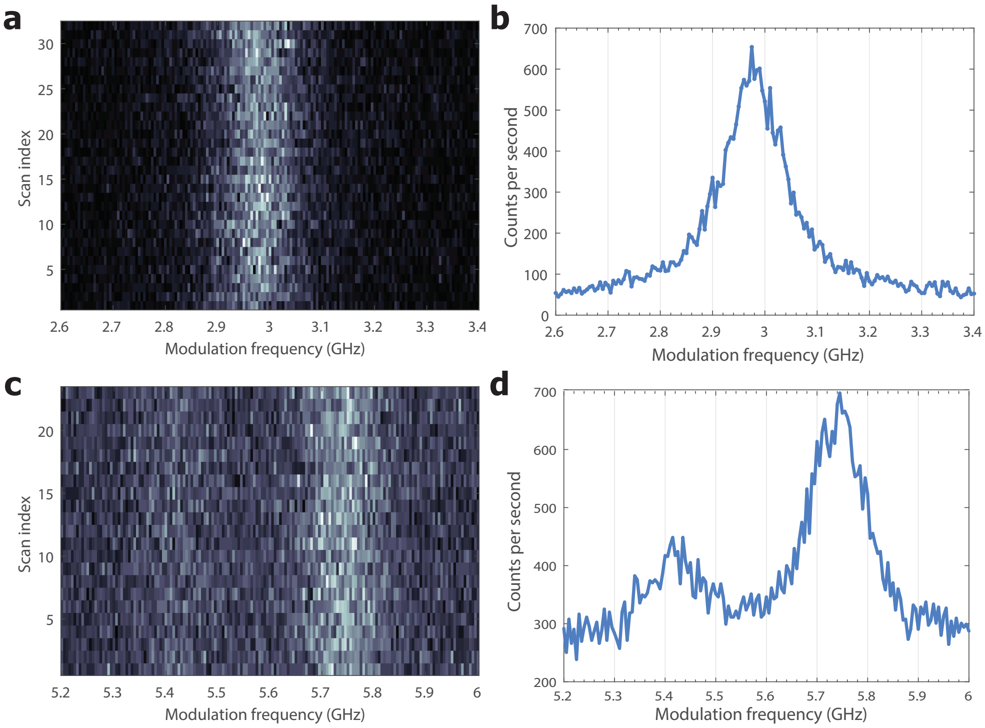

Supplementary Figure 14: (a) Continuous monitoring of the resonant excitation spectrum of the C transition at 0 V (ground state splitting of 88 GHz) and 0 T. Each horizontal line corresponds to a single acquisition of the spectrum over a duration of 0.6 s with 5 MHz spectral resolution. (b) Average of the 32 scans in part (a) corresponding to a total acquisition time of 20 s (c) Continuous monitoring of the resonant excitation spectrum of two of the four Zeeman-split C transitions at 400 V (ground state splitting of 303 GHz) and a B-field of 0.2 T. Each horizontal line corresponds to a single acquisition of the spectrum over a duration of 0.6 s with 5 MHz spectral resolution. (d) Average of the 23 scans in part (c) corresponding to a total acquisition time of 14 s.

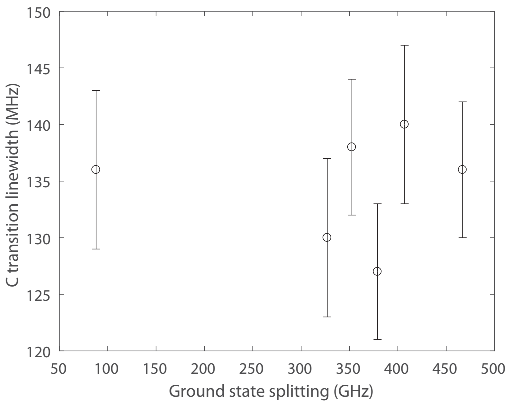

Supplementary Figure 15: Linewidth of the C transition measured at various strain conditions over a timescale of few minutes. Error bars are obtained from Lorentzian fits to the spectra.

Supplementary References

[1] Atikian, H. A. *et al.* Wafer-scale fabrication of freestanding nanostructures via reactive ion beam undercut etching (2017).

[2] Burek, M. J. *et al.* Free-Standing Mechanical and Photonic Nanostructures in Single-Crystal Diamond. *Nano Letters* **12**, 6084–6089 (2012).

[3] Sipahigil, A. *et al.* Supplementary materials - an integrated diamond nanophotonics platform for quantum-optical networks. *Science* **354**, 847–850 (2016).

[4] Schröder, T. *et al.* Scalable focused ion beam creation of nearly lifetime-limited single quantum emitters in diamond nanostructures. *arXiv:1610.09492 [quant-ph]* (2016).

[5] Chu, Y. *et al.* Coherent optical transitions in implanted nitrogen vacancy centers. *Nano Letters* **14**, 1982–1986 (2014).

[6] Evans, R. E., Sipahigil, A., Sukachev, D. D., Zibrov, A. S. & Lukin, M. D. Narrow-linewidth homogeneous optical emitters in diamond nanostructures via silicon ion implantation. *Phys. Rev. Applied* **5**, 044010 (2016).

[7] Maier, F., Riedel, M., Mantel, B., Ristein, J. & Ley, L. Origin of surface conductivity in diamond. *Phys. Rev. Lett.* **85**, 3472–3475 (2000).

[8] Chen, K. C.-Y. *et al.* Conductor fusing and gapping for bond wires. *Progress In Electromagnetics Research M* **31**, 199–214 (2013).

[9] Comsol multiphysics® v. 5.2. URL www.comsol.com.

[10] Zhan, S., Azarian, M. H. & Pecht, M. G. Surface insulation resistance of conformally coated printed circuit boards processed with no-clean flux. *IEEE transactions on electronics packaging manufacturing* **29**, 217–223 (2006).

[11] Townsend, J. S. (Oxford University Press, 1915).

[12] Strong, F. W., Skinner, J. L., Dentinger, P. M. & Tien, N. C. Electrical breakdown across micron scale gaps in mems structures. In *MOEMS-MEMS 2006 Micro and Nanofabrication*, 611103–611103 (International Society for Optics and Photonics, 2006).

[13] Meesala, S. *et al.* Strain engineering of the silicon vacancy center in diamond. *arXiV pre-print* arXiV 1801.09833 (2018).

[14] Sternschulte, H., Thonke, K., Sauer, R., Münzinger, P. C. & Michler, P. 1.681-eV luminescence center in chemical-vapor-deposited homoepitaxial diamond films. *Phys. Rev. B* **50**, 14554–14560 (1994).

[15] Hepp, C. *Electronic Structure of the Silicon Vacancy Color Center in Diamond*. Ph.D. thesis (2014).

[16] Jahnke, K. D. *et al.* Electron–phonon processes of the silicon-vacancy centre in diamond. *New Journal of Physics* **17**, 1–11 (2015).

[17] Neu, E. *et al.* Single photon emission from silicon-vacancy colour centres in chemical vapour deposition nano-diamonds on iridium. *New Journal of Physics* **13**, 025012 (2011).

[18] Hepp, C. Electronic structure of the silicon vacancy color center in diamond. *PhD dissertation* (2014).
